# Screening of *Ulva rigida*, *Gracilaria* sp., *Fucus vesiculosus* and *Saccharina latissima* as Functional Ingredients

**DOI:** 10.3390/ijms19102987

**Published:** 2018-09-30

**Authors:** Rodrigo T. Neto, Catarina Marçal, Ana S. Queirós, Helena Abreu, Artur M. S. Silva, Susana M. Cardoso

**Affiliations:** 1 QOPNA, Department of Chemistry, University of Aveiro, Campus Universitário de Santiago, 3810-193 Aveiro, Portugal; rodrigotrepaneto@gmail.com (R.T.N.); catarina.marcal@ua.pt (C.M.); queiros.as@gmail.com (A.S.Q.); artur.silva@ua.pt (A.M.S.S.); 2 Algaplus-Prod. e comercialização de algas e seus derivados, Lda, 3830-196 Ílhavo, Portugal; htabreu@algaplus.pt

**Keywords:** algae, antioxidant, α-glucosidase, lipase, angiotensin-I converting enzyme, chemical composition

## Abstract

The intent of the present work was to evaluate the potential of four macroalgae prevalent in Europe, namely *Ulva rigida*, *Gracilaria* sp., *Fucus vesiculosus* and *Saccharina latissima*, for application in functional foods, either in the direct form or as extracts. Accordingly, nutritional composition, the content of phytochemical antioxidants, and the inhibitory ability of key enzymes with impacts on obesity and diabetes (α-glucosidase and pancreatic lipase) or on arterial pressure (angiotensin-I converting enzyme), were evaluated. Overall, protein, lipid, ash and fiber contents of the macroalgae ranged from 9–24% dw, 0.5–3.0% dw, 20–32% dw, and 37–45% dw, respectively, making them good candidates for nutritional supplementation of several foods, particularly due to their mineral and fiber contents. In addition, brown macroalgae, in particular *F. vesiculosus,* stood out for its superior phenolic content, which was reflected by its high antioxidant ability and inhibition towards α-glucosidase activity (0.032 mg/mL of hydroacetonic extract inhibited 50% of the enzyme activity).

## 1. Introduction

In the last few decades, society has seen a dramatic increase in the incidence of cardiovascular diseases (CVD) such as coronary heart disease, and metabolic disorders (MD) such as diabetes. It has been estimated that by 2030, approximately 23.6 million people will die from CVD [1] and more than 640 million will have diabetes by 2040 [2]. Despite the contribution of genetic factors to both types of pathologies, they are largely influenced by an unhealthy diet (specifically the high intake of saturated fats and carbohydrates, respectively), physical inactivity, tobacco consumption and obesity [3]. Consequently, several initiatives have been developed to increase public awareness of the preemptive role of nutrition in the mitigation of these disorders. Furthermore, for the last several years, there has been an increase in concerns regarding the safety of synthetic food additives (e.g., antioxidants) and their long-term effects on human health, specifically relating to their mutagenic and carcinogenic effects [4]. All these concerns have prompted an increased demand in the search for natural alternatives that can be applied as food or as ingredients in food formulations with the aim of improving their nutritional or functional values, or of replacing synthetic additives used in the biological and chemical stabilization of food.

Seaweeds, that is, marine macroalgae (including Chlorophyta, Rhodophyta and Ochrophyta/Phaeophyceae), are considered one of the non-animal foods of the future due to their ability to grow without using arable land or fresh water resources, thus not competing with traditional crops. They are also a rich and balanced source of nutrients and bioactive phytochemicals, of which regular consumption has been correlated with the prevention of several diseases [5]. Although the direct consumption of macroalgae as food is still incipient in Europe when compared to the Asiatic countries, there is a trend towards increasing consumption of these “sea vegetables” [6]. Meanwhile food and nutraceutical industries also have a growing interest in introducing macroalgae as ingredients in functional foods, and this combined effect is boosting macroalgae as part of dietary habits in European countries. Macroalgae are particularly attractive due to their richness in dietary fibers, minerals, proteins, steroids and phenolic compounds, which in turn are closely related to the claimed health properties [7].

Dietary fiber from seaweeds represents 25% to 75% of their dry weight and consists mostly of carrageenan and agar (red algae), alginates (brown algae) and ulvans (green algae) [8]. As for dietary fibers in general, they exhibit several health benefits, mainly due to their oil and water holding capacities. The latter in particular can improve the viscosity of newly formulated food products, besides promoting their absorptive capacity and fecal bulking capacity and fermentability in the alimentary canal [9]. In parallel, the mineral fraction of seaweed has also received a lot of attention since it can reach up to 40% of seaweed’s dry weight [10]. In addition, many seaweeds show a high potassium/sodium ratio that can help to balance modern dietary habits, which are typically characterized by the intake of high and unhealthy levels of sodium [11]. Moreover, red and green macroalgae are painted as an alternative, or complement, to traditional protein sources, as levels in some seaweed species can reach up to 47%and 26% of the dry weight (dw) [12], respectively.

Phlorotannins represent the most noticeable seaweed phenolic compounds. These are found in high concentrations in brown algae (up to 25%), resulting from the polymerization of phloroglucinol and its derivatives. These compounds show high antioxidant activity both as pure compounds [13] and as crude extracts [14], and have been successfully employed as a food additive [15]. In addition, phlorotannins have been shown to exert a number of other biological activities, including the inhibition of α-amylase, α-glucosidase and lipase, which are key enzymes in obesity and diabetes control, as well as angiotensin converting enzyme (ACE); i.e., a specific target for hypertension management [16,17].

More recently, several scientific articles have been published concerning the nutritional and health-promoting characteristics of seaweed species that are common in Europe. Among chlorophyta, *Ulva rigida* was characterized for its high ash (20.6%) and low lipid (1.02%) content [18], in addition to being capable of effectively inhibiting ACE activity [19]. Another example is the phaeophyceae seaweed, *Fucus vesiculosus*, that has been described as having high Ca (1.16%) and Fe (0.019%) contents [20], and being a good raw material for extracts presenting high antioxidant [21] and enzyme inhibition [22,23] activities. *Saccharina latissima*, on the other hand, despite having its chemical composition well characterized in several studies, with special emphasis being given to its high ash content (43.8%) and overall mineral [24] and amino acid [25] profiles, remains poorly described as an enzyme inhibitor. Finally, the genus *Gracilaria*, belonging to rhodophyta, has been studied both for its composition, especially its high saccharide content [26,27], and for the ability of its polysaccharides to inhibit lipase activity, with studies undertaken both in vivo [28,29] and in vitro [30,31].

Despite the considerable amount of information concerning the beneficial effects of specific macroalgae molecules [32], these have generally been tested after extensive purification procedures that are not expected to be applied in the food industry due to their associated costs. Hence, this work sets out to highlight the potential of four macroalgae prevalent in Europe, namely *Ulva rigida*, *Gracilaria* sp., *Fucus vesiculosus* and *Saccharina latissima*, for application as food ingredients in functional foods, or as supplements, either in the direct form or as extracts. Accordingly, nutritional composition, the content of phytochemical antioxidants and the inhibitory ability of key enzymes with impacts on obesity and diabetes (α-glucosidase and pancreatic lipase) or on arterial pressure (angiotensin-I converting enzyme) were assessed. To the best of our knowledge, this is the first study that analyzes seaweeds and their extracts in a holistic way instead of just focusing on one specific aspect, demonstrating that the same seaweed/extract can be used in the food industry to improve nutritional value and contribute to reducing the impact of pathologies associated with the modern life style. Also, contrary to most previous studies, which focused on wild macroalgae, the present study has been undertaken with macroalgae cultivated under controlled and sustainable cultivation systems; i.e., in land-based or sea long-lines which are believed will have a central role in supplying sufficient biomass levels to meet market development needs in coming years.

## 2. Results

### 2.1. Seaweeds’ Nutritional Characterization

The selected seaweeds were particularly rich in carbohydrates (46.9–68.9 g/100 g dw, Table 1), with fibers representing the major contributors (36.6–45.0 g/100 g dw, corresponding to 59–85% of carbohydrate content). Notably, the two brown macroalgae, *F. vesiculosus* and *S. latissima* stood out for their high amounts of insoluble fiber compared to soluble fiber, whereas the soluble/insoluble fiber ratio in red and green seaweeds was close to 1.

As expected, the mineral content of the four macroalgae was also high, ranging from 20.4 ± 0.1 g/100 g dw in *S. latissima* to 31.7 ± 0.6 g/100 g dw in *U. rigida*. All samples had high K levels as well as K/Na ratios above one, with *Gracilaria* sp. presenting the most interesting value (5.8), besides other important minerals that were also detected in considerable amounts. Among these, the two brown seaweeds were particularly interesting for their Ca and Mg amounts, that reached a maximum of 1382.0 ± 5.1 mg/100 g dw and 835.5 ± 9.7 mg/100 g in *F. vesiculosus*, respectively. Notably, this latter mineral was also particularly accumulated in *U. rigida*, where it accounted for 3758.6 ± 430.3 mg/100 g dw. Furthermore, *Gracilaria* sp. and *S. latissima* stood out for their Fe levels (8.5 and 9.7 g of these seaweed respectively, fill its reference daily intake).

In addition to carbohydrates and minerals, the four macroalgae also showed moderate to high protein contents, that were particularly evident in *Gracilaria* sp. (23.6 ± 0.2 g/100 g dw). In turn, as previously described for seaweeds in general [33], their lipid fraction was low, ranging from 0.5 ± 0.1 g/100 g dw in *S. latissima* to 3.0 ± 0.3 g/100 g dw in *F. vesiculosus*. Globally, the high fiber and low-fat levels resulted in a low caloric value: 167.3, 112.7, 162.6 and 198.3 kcal/100 g dw for *U. rigida*, *Gracilaria* sp., *F. vesiculosus* and *S. latissima*, respectively.

Despite the lipid content of macroalgae being in general low, their lipid profile is often highlighted as being rich in polyunsaturated fatty acids, and hence might be of interest in food supplementation [12]. The content of relevant lipid components from the four seaweeds is summarized in Table 2. Polyunsaturated acids were abundant and consequently, the lowest unsaturated/saturated ratio was about 0.8. The unsaturated fraction in the four algae was mainly composed of two monounsaturated fatty acids (palmitoleic and oleic acid), along with the polyunsaturated fatty acids, linoleic acid (an Ω6) and α-linolenic plus eicosapentanoic acids (Ω3). Notably, with the exception of *U. rigida*, the polyunsaturated fraction predominated over the monounsaturated one, representing 66%, 76% and 79% of the unsaturated fatty acids in *Gracilaria* sp., *F. vesiculosus* and *S. latissima*, respectively. Overall, the Ω6/Ω3 ratio was below 0.5 (0.36–0.48) in *U. rigida*, *F. vesiculosus* and *S. latissima*, and was particularly low in *Gracilaria* sp. (0.22). On the other hand, regardless of the sample, palmitic acid was the most abundant saturated fatty acid, representing roughly half of the total saturated fraction. In addition, *F. vesiculosus* showed relevant levels of myristic and stearic acids, which amounted for 927.9 ± 114.6 and 539.9 ± 37.9 mg/kg dw, respectively.

In addition to fatty acids, the lipid fraction of the four algae also contained considerable amounts of butyl 6,9,12,15-octadecatetraenoate (a fatty acid ester), 2-hexadecen-1-ol (a long chain alcohol), phytol and neophytadiene (diterpenes) and cholesterol derivatives. Interestingly, *U. rigida* and *F. vesiculosus* had close amounts of butyl 6,9,12,15-octadecatetraenoate (160.8 ± 42.2 and 182.4 ± 14.1 mg/kg seaweed dw) and neophytadiene (172.3 ± 7.6 and 182.5 ± 35.3 mg/kg seaweed dw, respectively), despite the higher lipid levels of *F. vesiculosus*. Another interesting observation was the high phytol content observed in *Gracilaria* sp. samples (279.7 ± 24.8 mg/kg dw), even though its lipid fraction only accounted for 0.7 g/100 g dw. Furthermore, its total content of cholesterol derivatives was also very high (888.1 mg/kg dw).

### 2.2. Seaweeds’ Water and Oil Holding Capacities

**Figure 1:**
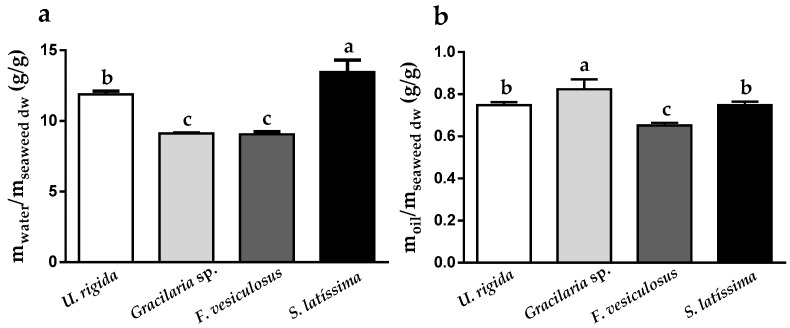
(**a**) water, and (**b**) oil holding capacity of *Ulva rigida*, *Gracilaria* sp., *Fucus vesiculosus* and *Saccharina latissima*. Different letters in the same graph indicate significant differences (*p* < 0.05).

Water and oil holding capacities (WHC and OHC) are believed to be key factors in the beneficial properties of fibers. Overall, the results indicated that the WHC of the four samples ranged from 9.06 (*F. vesiculosus*) to 13.47 g/g seaweed dw (*S. latissima*) (Figure 1a). Notably, the two brown algae were clearly distinguishable with regard to their WHC and OHC functions, despite their similar fiber content (45.0 ± 0.1 and 40.9 ± 0.6% dw, respectively). This is even more relevant when considering the fact that the procedure followed for WHC determination (incubation at 37 °C for 24 h followed by centrifugation) mainly considers the insoluble seaweed fraction [34]. Hence, one might conclude that although it has a much lower insoluble fiber content (28.2 g/100 g seaweed dw, Table 1) in comparison to *F. vesiculosus* (37.4 g/100 g seaweed dw, Table 1), *S. latissima* is able to hold 50% more water (Figure 1a). Following the same trend, *U. rigida* also showed enhanced WHC when compared to *F. vesiculosus*, regardless of its lower fiber content (total and insoluble fiber levels of 36.6 ± 1.5% and 18.9 ± 0.8% dw vs. 45.0 ± 0.1% and 37.4 ± 0.3% dw, respectively). On the other hand, the OHC of the four macroalgae was much lower than the WHC and differences in their behavior were subtle, with values ranging from 0.65 (*F. vesiculosus*) to 0.82 (*Gracilaria* sp.) g/g seaweed dw (Figure 1b).

### 2.3. Phenolic Content and Antioxidant Activity of Seaweed Extracts

The use of extracts in the food and nutraceutical industries is based on their functionalities, with antioxidant activity being one of the most commonly exploited, given its importance in counteracting/preventing oxidative stress events that underlie numerous pathological conditions such as inflammatory diseases, or even obesity and diabetes. In the present work, the antioxidant activity of the samples was evaluated by 2,2-diphenyl-1-picrylhydrazyl (DPPH) and ferric reducing antioxidant power (FRAP) methods and the results were expressed as grams of ascorbic acid and butil-4-hidroxianisol equivalents (AAE and BHAE, respectively) per 100 g of extract, respectively.

As shown in Table 3, the yield of extraction of aqueous extracts was higher than those obtained with organic solvents. This was particularly relevant for *U. rigida* and *F. vesiculosus*, for which aqueous extracts represented 43–46% (*w*/*w*) and 28–31% (*w*/*w*), respectively, while organic extracts accounted for 15–19% (*w*/*w*) in both algae. Moreover, it was also possible to conclude that mass recovery of extracts obtained at room temperature (RTWE) was generally lower than those obtained with hot water extraction (HWE).

The total phenolic (TPC) content has been described as one of the most relevant factors associated with several biological activities of seaweeds and/or their extracts. Despite lower yields, 80% (*v*/*v*) ethanol and 70% (*v*/*v*) acetone extracts (Eth80 and Acet70, respectively) showed higher TPC contents, except for *S. latissima* (HWE was the richest extract). This fact was particularly noticed in *F. vesiculosus*, where TPC accounted for 5.66 and 3.91 g gallic acid equivalents (GAE)/100 g extract in Eth80 and Acet70, respectively (Table 3) and only 1.5–1.7 g GAE/100 g extract in the aqueous extracts. In parallel, Eth80 and Acet70 from *F. vesiculosus* exhibited the highest antioxidant abilities, as measured by the DPPH (2.60 and 1.47 g AAE/100 g extract, respectively) and FRAP methods (8.30 and 8.03 g BHAE/100 g extract, respectively). The data gathered allowed us to conclude that overall *F. vesiculosus* was the most promising macroalgae with respect to TPC and antioxidant abilities, with better results being achieved even in aqueous extracts when compared to organic and aqueous extracts of the remaining algae samples.

### 2.4. Enzyme Activity Inhibition by Seaweed Extracts

The enzyme α-glucosidase is an important therapeutic target enzyme due to its involvement in the digestive process stage of complex dietary carbohydrates, where it metabolizes simpler oligosaccharides for intestinal absorption. Thus, inhibition of this enzyme, besides being able to retard the digestion of oligosaccharides and disaccharides, also delays the absorption of glucose by the small intestine and reduces plasma glucose levels. In addition, pancreatic lipase and ACE are also therapeutic targets in the treatment of obesity and hypertension, respectively. Lipase inhibition reduces the levels of free fatty acids and monoacylglycerols in the intestinal lumen, contributing to a decrease in the absorption of triacylglycerols, whereas the inhibition of ACE blocks the effect of angiotensin II leading to relaxation of the blood vessels, thus inducing blood pressure reduction [35].

The inhibitory potential of the extracts towards the above-mentioned enzymes varied considerably. Indeed, those from brown seaweeds were able to inhibit α-glucosidase with varying levels of effectiveness (Table 4). Amongst them, *F. vesiculosus* extracts were significantly more effective than those of *S. latissima*, which is probably related to their superior content of phenolic compounds/phlorotannins, since these are known to counteract α-glucosidase [36]. Notably, the IC_50_ (extract concentration able to inhibit the activity of the enzyme by 50%) of Acet70 from *F. vesiculosus* was about eight-fold less than that of acarbose (IC_50_ 0.032 ± 0.003 vs. 0.264 ± 0.041 mg/mL, respectively), which is a specific drug for this enzyme. Moreover, despite its low phenolic content as compared to *F. vesiculosus* Acet70, the RTWE from the same macroalgae also exhibited high inhibitory activity against α-glucosidase, suggesting that non-phenolic components in this extract may also play a relevant role in the inhibition of this enzyme.

On the other hand, the data gathered allowed us to conclude that in general, extracts of *U. rigida*, *S. latissima*, *F. vesiculosus* or *Gracilaria* sp. origin only exerted modest inhibition towards lipase and/or ACE. Five of the tested extracts, namely RTWE, HWE and Acet70 (from *F. vesiculosus*), Eth80 from *Gracilaria* sp. and *S. latissima* were able to counteract the activity of lipase, for 15–31% at 3 mg/mL, while those of *U. rigida* were completely ineffective up to that concentration. These latter in turn, along with those of *Gracilaria* sp. and *S. latissima,* were able to impair the activity of ACE. The most relevant extracts against this enzyme were Eth80 and Acet70 from *Gracilaria* sp. (IC_50_ of 3.21 ± 0.23 and 3.45 ± 0.26 mg/mL, respectively), followed by the organic extracts from *U. rigida* and *S. latissima* (IC_50_ in the range of 3.67–4.56 mg/mL) and RTWE from *S. latissima* (IC_50_ 4.48 ± 0.66 mg/mL). These results suggest that the activity of these crude extracts would probably be modest if used directly as an ingredient in food industry. Still, it is noted that their purification might be of interest in the search for purified molecules with potential activity toward pancreatic lipase and/or ACE with applications in pharmacy or medicines.

### 2.5. Seaweed Extracts’ Nutritional Characterization

Considering the economic and the financial constraints that normally occur in the food industry, we selected the aqueous extracts for further characterization regarding their nutritional values (Table 5). Ash was a major component in all extracts, ranging from 28 to 56% (*w*/*w*) of extract dw and was particularly high in those from green and red algae-origins, which in part is consistent with the high ash levels found in *U. rigida* and *Gracilaria* sp. (29–32% dw, Table 1). In addition, *Gracilaria* sp. RTWE also had high protein levels, which can be explained by the high protein content of *Gracilaria* sp. (23.6 ± 0.2% dw, Table 1), presumably corresponding to water-soluble phycobiliproteins [37]. In turn, regardless of the high fiber content of *Gracilaria* sp., their solubilization in RTWE was significantly low if compared to the same extracts from the remaining algae. This was particularly evident for *F. vesiculosus* and *S. latissima,* since despite their low soluble fiber content (7.6 ± 0.3 and 12.8 ± 0.3% dw, respectively) levels in the RTWE accounted for 17–25% extract dw. Also note that particularly in these two macroalgae, the recovery of carbohydrates was significantly increased in HWE with respect to RTWE, even though the extraction time was considerably less. In both algae, the soluble fiber content of HWE was enriched by about 8%, while free sugars, expressed as glucose equivalents (GlcE), increased from 7.5 ± 1.0 to 13.8 ± 2.1% GlcE dw in *F. vesiculosus*, and from 18.2 ± 0.7 to 23.4 ± 3.1% GlcE dw in *S. latissima*).

## 3. Discussion

In general, the nutritional values of *U. rigida* were in accordance with those previously reported for wild samples from other regions, including the Azores and Tunisia ([18,38]), for which protein and ash contents ranged from 7 to 16% dw and 21 to 26% dw, respectively. Moreover, the results herein obtained for *Gracilaria* sp. were within the range normally found in common species of the *Gracilaria* genus; specifically, protein content (5 to 23% dw), lipid content (0.4 to 2.8% dw), total carbohydrate content (15 to 63% dw) and ash content (8 to 53.3% dw) [26,39]. The lipid content of *F. vesiculosus* was within the limits previously reported (2.65–8.4% dw) [40], although the protein and ash contents were slightly higher than the values reported by the same authors (12.2 and 22.5% dw, respectively). The nutritional profile of *S. latissima* was in line with the one previously published by Tibbetts et al. [40]. Discrepancies can however occur due to the use of different extraction solvents in crude lipid extraction (e.g., chloroform/methanol instead of light petroleum), and/or differences regarding seasonal and geographic factors. Our water holding capacity values are slightly higher than the ones reported by other authors, which might be related to experimental differences, as in this study the solubilization of compounds was considered, therefore resulting in higher values [27,41]. The oil holding capacity values were determined with the same experimental procedure and are more in line with those of the cited authors.

It is noted that the most exploited area of food application for seaweeds has been in meat products. Their implementation has already been shown to improve meat product characteristics; namely, animal fat decrease, water and oil retention increase, salt reduction in product formulation, and increased mineral and dietary fiber content. These aspects have been described by Fernández-Martín et al. [42] and López-López et al. [43] with the addition of *Himanthalia elongate* powder to pork batter and frankfurters respectively, by Kim et al. [44] with the addition of *Lamina japonica* to breakfast sausages, and by López-López et al. [45] with the addition of *Undaria pinnatifida* to beef patties. In the articles mentioned, seaweed powder was used in concentrations as high as 5% (*w/w*) that in some cases resulted in negative organoleptic characteristics. Nevertheless, a consistent increase of about 50% in mineral content (increase in Ca, Mg and K) and emulsion stability were verified.

Our results demonstrated that, as in the above studies, the macroalgae *U. rigida*, *Gracilaria* sp., *F. vesiculosus* and *S. latissima* typically found in Europe, can be employed in the food industry as a replacement for other additives. The high levels of soluble fiber in *U. rigida* and *Gracilaria* sp., as well as the high WHC ability of *S. latissima* insoluble fiber, make these algae more suitable for increasing water/oil retention in meat products, while *Gracilaria* sp. is the most appropriate one when the intention is to impact on the final protein composition of newly formulated products. In addition, *Gracilaria* sp. has a high K content and K/Na ratio (Table 1), ideal for sodium chloride replacement. The high content of other minerals can also be considered (Table 1); i.e., Ca and Mn in *F. vesiculosus*, Mg in *U. rigida*, Fe in *Gracilaria* sp. and *S. latissima*. Mineral content is even more important when considering water-based extracts that have an increased ash content (29–56% extract dw, Table 5) and can be used in lower percentages, with higher impact on the mineral profile of the new products.

The previous use of seaweed-based extracts has been tested by several authors, mainly to increase food functionalities that are associated with antioxidant capacity. In this regard, Moroney et al. [46] showed the ability of extracts containing fucoidan and laminarin from *Laminaria digitata* to act as a natural antioxidant at a concentration of 6 mg/mL, despite not being as effective as synthetic extracts. On the contrary, Jónsdóttir et al. [47] and Wang et al. [21] showed the potential of *F. vesiculosus* phlorotannin-rich extracts to inhibit lipid oxidation in minced cod at a concentration of 300 mg/kg of cod, while Ortiz et al. [48] demonstrated the use of aqueous extracts of several seaweeds as covering solution for the chemical preservation of canned salmon. Among the four seaweeds focused on in this study, the results highlighted the high antioxidant potential of *F. vesiculosus* (Table 3), which is probably associated with the presence of phlorotannins in the extracts and may be indicative of its potential to function as a replacement for synthetic additives/antioxidants. In addition, one should not disregard the abilities of some of the tested extracts towards specific enzymes, with relevance for specific health disorders. The results indicated, firstly, the potential of *F. vesiculosus* extracts to strongly inhibit α-glucosidase, especially Acet70 (IC_50_ = 32 µg/mL) and Eth80 (IC_50_ = 119 µg/mL). This allows for its use in the management of MD such as diabetes, by controlling glycemic levels, and effectively reducing the assimilable caloric content of foods thus lowering the caloric content without changing their formulation, besides the addition of the extract. Our results are in accordance with previous studies that analyzed the effect on α-glucosidase of *F. vesiculosus*’ fucoidans [22] with an IC_50_ > 50 mg/mL and commercially-available polyphenol extracts [49,50], with IC_50_ of 0.604 µg/mL and 5 µg/mL, respectively, when obtained with hot water but further purified with ultrafiltration. Certain molecules from other seaweed species have also been studied for their ability to inhibit α-glucosidase, particularly, a triterpenoid from *Padina boergesenii* [51], a triterpenic acid from *Codium dwarkense* [52], octaphlorethol A from *Ishige foliacea* [53] and crude extracts from *Grateloupia elliptica* [54], with IC_50_’s of <100 µg/mL, 3.31 µg/mL, 109 µg/mL and 20 µg/mL, respectively. However, these compounds were again obtained through extensive purification procedures with organic solvent extraction and column fractionation. To the best of our knowledge, this is the first study estimating the ability of organic and aqueous extracts from *S. latissima* origin in modulating α-glucosidase activity. As indicated, the best activity in this case was achieved for the Acet70 extract (IC_50_ = 1.68 mg/mL), possibly partly associated with the presence of phlorotannins.

Similarly, some of the analyzed extracts showed the ability to partially inhibit the activity of lipase. Despite this effect only being visible at higher concentrations (3 mg/mL) and hence possibly not effective if used as a food ingredient, this information might signal the potential for Eth80 extracts from *S. latissima* and *Gracilaria* sp., or aqueous extracts from *F. vesiculosus*, to serve as a valuable source of molecules for the control of lipase activity. To the best of our knowledge this is the first study evaluating the ability of organic and aqueous crude extracts from *U. rigida*, *Gracilaria* sp., *F. vesiculosus* and *S. latissima* towards lipase activity and might consolidate previous in vivo studies. For example, Vázquez-Freire et al. [23] showed that a polysaccharide extract from *F. vesiculosus* could exert hypolipidaemic activity in rats. Also, it has been reported that feeding hyperlipidaemic rats with powdered *Gracilaria changii* [28,29] or *Saccharina japonica* [55] could be used to treat this type of pathology, by improving lipid metabolism and increasing lipolitic enzyme activity. In addition, some authors have described the direct interaction of lipase with commercial alginates [30,31] (IC_50_ > 3 mg/mL) and ethanol extracts of *Kappaphycus alvarezii*, *Kappaphycus striatus* and *Eucheuma denticulatum* [56] (inhibition percentages varying from 83 to 92% at 3.8 mg/mL), further validating the use of algae as a possible source of compounds for the treatment of lipid metabolic disorders and related health complications.

As far as the inhibition of ACE is concerned, our findings also showed that crude extracts from *U. rigida*, *Gracilaria* sp. and *S. latissima* could partially inhibit its activity, although for an effective in vivo control of high blood pressure, purified fractions would probably be required. This data is consistent with the observations of Paiva et al. [19] for wild *Ulva rigida* extracts (IC_50_ water extract = 0.483 mg/mL; IC_50_ of peptide purified fraction = 0.069 mg/mL). Note that to the best of our knowledge there are no previous studies testing the inhibitory potential of *Gracialria* sp. or *S. latissima* on ACE.

## 4. Materials and Methods

### 4.1. Chemicals

Nitric acid, ethanol, acetone, isopropanol, sodium carbonate decahydrate and sodium phosphate monohydrate were purchased from Panreac (Barcelona, Spain). Light petroleum, pyridine, trichloroacetic acid, sodium chloride, potato starch, potassium and sodium tartrate tetrahydrate, sodium hydroxide and tris base were purchased from Fisher. Tetracosane, 2,2-diphenyl-1-picrylhydrazyl (DPPH), Folin-Ciocalteu reagent, α-glucosidase from *Saccharomyces cerevisiae*, 4-nitrophenyl α-d-glucopyranoside (pNPG), lipase from porcine pancreas, colipase from porcine pancreas, 1,2-di-*O*-lauryl-rac-glycero-3-(glutaric acid 6-methylresorufin ester) (DGGR), angiotensin converting enzyme from rabbit lung (ACE) and *N*-[3-(2-furyl)acryloyl]-phe-gly-gly (FAPGG) were purchased from Sigma (St. Louis, MO, USA). N,O-bis(trimethylsilyl)trifluoroacetamide (BSTFA), cholesterol, palmitic acid, 3,5-dinitrosalicylic acid, sodium deoxycholate, sodium taurodeoxycholate hydrate and captopril were purchased from Acros (Hampton, NH, USA). Pentadecanol and Orlistat were purchased from TCI (Tokyo, Japan). Sodium acetate and calcium chloride were purchased from ChemLab (Eernegem, Belgium). Acarbose was purchased from Fluka (Bucharest, Romania). All reagents were of analytical grade or of the highest available purity.

### 4.2. Seaweeds

*Gracilaria* sp., *Fucus vesiculosus*, *Ulva rigida* and *Saccharina latissima* were provided by ALGAplus Lda, a company based in the Aveiro district, Portugal, specializing in the production of seaweeds in land-based aquaculture and their commercialization in the food and cosmetic markets. The first three algae species were produced at ALGAplus site under a land-based Integrated Multi-Trophic Aquaculture and collection was made in March, July and January of 2016, respectively, while *Saccharina latissima* was cultivated by a seaweed aquaculture in Northern France (Brittany), under sea long-lines and harvested in April of 2015. All seaweeds were dried by the companies following internal procedures for commercialization purposes, using mild air current drying at maximum of 30 °C. Samples were kept in a dry, dark and fresh place at room temperature until needed.

### 4.3. Chemical Characterization

#### 4.3.1. Proximate Composition

The chemical composition of samples was determined according to AOAC methods [57]. Total protein content was estimated by determination of elemental nitrogen content by thermal conductivity using a TruSpec 630-200-200 CNHS analyzer from LECO (St. Joseph, MI, USA), multiplied by the conversion factor of 5, which is specific for seaweeds [58]. Ash content was determined by incineration in a muffle furnace at 550 °C for 6 h and gravimetric quantification. Crude fat was obtained by soxhlet extraction with light petroleum for 8 h, followed by filtration through a 0.2 µm nylon filter, solvent removal in a rotary evaporator and drying, followed by gravimetric quantification. Total carbohydrates were calculated by difference. Dietary fiber contents (soluble, insoluble and total) were estimated according to the enzymatic gravimetric method AOAC 991.43, using the Total Dietary Fiber Assay kit from Megazyme (Bray, UK). Calorific content was determined according to AOAC [57]. The free sugars content was measured according to Dubois et al. [59] with slight modifications. In particular, 50 µL of 4% phenol and 250 µL of 96% sulfuric acid was added to 100 µL of sample/standard. The absorbance of the mixture was read at 490 nm after 10 min of incubation.

#### 4.3.2. Mineral Composition

Approximately 1 g of ash was digested with three additions of 10 mL of concentrated nitric acid. After digestion, samples were filtered through a no. 4 paper filter and their volume was adjusted to 100 mL with ultrapure water. Elements (Na, K, Ca, Mg, Fe, Zn, Mn, Cu and Ni) were quantified in a Perkin Elmer (Waltham, MA, USA) Analyst 100 flame atomic absorption spectrometer equipped with single hollow cathode lamps for each element and an air-acetylene burner.

#### 4.3.3. Lipidic Profile

Approximately 20 mg of crude fat extract was mixed with 250 µL of internal standard (1.5 mg/mL of tetracosane dissolved in pyridine), followed by the addition of 250 µL of *N*,*O*-bis(trimethylsilyl)trifluoroacetamide (BSTFA) and 50 µL of trimethylchlorosilane (TMSCl). Silylation was achieved by incubation at 70 °C for 30 min and lipid characterization was performed in a Gaseous Chromatographer coupled with a Mass Spectrometer (GC-MS), GCMS-QP2010 (Shimadzu (Kyoto, Japan)) equipped with an AOC-20i auto-injector and a DB-5 ms column (30 m × 0.25 mm diameter, 0.25 µm thickness). The elution program was the same as described elsewhere [60]. This started with 70 °C for 5 min followed by a temperature increase of 4 °C/min until 250 °C was achieved and a 2 °C/min increase until 300 °C, which was maintained for 5 min. The injection temperature was 320 °C and the split ratio was 100:0. Quantitative analysis was performed resorting to pure reference compounds (cholesterol, pentadecanol and palmitic acid), representative of the samples’ major lipophilic families.

### 4.4. Seaweeds’ Water and Oil Holding Capacity

For water and oil holding capacity, a method described elsewhere was used [61]. Briefly, 200 mg of dried and ground seaweed (*m_s_*) were incubated with 10 mL of deionized water or food oil in a previously weighted tube (*m_t_*) for 24 h at 37 °C in an orbital shaker, followed by centrifugation at 6000× *g* for 30 min and supernatant removal. For water holding capacity (WHC), samples were weighted (*m*_1_), frozen, lyophilized and weighted again (*m*_2_) and WHC estimated by Equation (1). For oil holding capacity (OHC), determination was made with Equation (2), where samples were weighted (*m*_3_) after centrifugation and supernatant removal.

(1) $$\;WHC=\frac{m_{1}-m_{2}}{m_{2}-m_{t}}\;$$

(2) $$\;OHC=\frac{m_{3}-m_{s}-m_{t}}{m_{s}}\;$$

### 4.5. Extract Preparation

Seaweeds (ground and sieved through a 0.224 mm pore) were subjected to four different extraction procedures using the following solvents and conditions, in a proportion of 1:20 (m:v): (i) deionized water at room temperature for 24 h; (ii) deionized water at 90 °C for 30 min; (iii) 80% (*v*/*v*) ethanol for 24 h at room temperature and (iv) 70% (*v*/*v*) acetone for 24 h at room temperature. Note that *Gracilaria* sp. samples was not extracted with deionized water at 90 °C due to its high agar content, which interferes with the subsequent downstream filtration steps. After extraction, all samples were centrifuged at 6000× *g* for 10 min and filtered through a G4 glass filter, followed by solvent removal in a rotary evaporator at 40 °C. Extracts were then frozen, lyophilized and stored at 4 °C until use.

### 4.6. Quantification of Phenolic Content of the Extracts

Total phenolic content was determined using Folin-Ciocalteu by the general methodology previously reported by Catarino et al. [62], with minor adaptations. Briefly, in a 96-well plate 15 µL of Folin-Ciocalteu reagent was added to 50 µL of deionized water followed by the addition of 15 µL of sample/standard and 5 min of incubation at room temperature. After that, 150 µL of 7% (*w*/*v*) sodium carbonate solution was added to each well and the plate was incubated for 60 min at 30 °C. The absorbance was measured at 750 nm.

### 4.7. Extracts’ Antioxidant Activity

The antioxidant activity of the extracts was estimated by two distinct methods; namely, 2,2-diphenyl-1-picrylhydrazyl (DPPH) [46] and ferric reducing antioxidant power assays (FRAP) [63], following the previously described methodology, with some modifications. Briefly, 250 µL of 8.66 × 10^−5^ M DPPH solution was added to 50 µL of sample/standard in a 96-well plate and incubated in the dark for 30 min. The absorbance was measured at 517 nm and ascorbic acid was used as standard. For FRAP assays, 200 µL of phosphate buffer (200 mM, pH 6.6) was added to 200 µL of sample and 200 µL of 1% (*w*/*v*) solution of potassium hexacyanoferrate. The mixture was then incubated at 50 °C for 20 min and 200 µL of 10% (*w*/*v*) trichloroacetic acid solution was added. Absorbance was measured at 690 nm and 3-tert-butyl-4-hydroxyanisole (BHA) was used as standard.

### 4.8. Ability of Seaweed Extracts to Inhibit Key Metabolic Syndrome Relevant Enzymes

#### 4.8.1. α-Glucosidase Activity

The activity of α-glucosidase was measured according to Sarkar et al. [64], with slight modifications. 50 µL of the solubilized extract (in phosphate buffer 50 mM, pH 6.8) was mixed with 50 µL of 6 mM *p*-nitrophenyl α-d-glucopyranoside (pNPG), dissolved in deionized water, followed by the addition of 100 µL of α-glucosidase solution. The plate was incubated for 20 min at 37 °C and the absorbance was measured every 60 s at 405 nm. Acarbose was used as a positive control of inhibition.

#### 4.8.2. Pancreatic Lipase Activity

The lipase activity was measured according to the procedure described by Panteghini et al. [65], with slight modifications. The reaction mixture was prepared in a microtube by mixing 55 µL of extract (dissolved in Tris buffer 50 mM, pH 8.4) with 385 µL of solution A (Tris buffer 50 mM, pH 8.4, containing 1.8 mM of sodium deoxycholate and 1.0 µg/mL of colipase) and 82.5 µL of solution B (acetate buffer (18 mM, pH 4.5):isopropanol (3:2) (*v*/*v*) with 0.2 mM of calcium chloride, 72 mM of sodium taurodeoxycholate and 136 µM of DGGR). The reaction was started by adding 27.5 µL of lipase solution and quickly transferred to a 96-well plate. The plate was incubated for 35 min at 37 °C and the absorbance was measured every 60 s at 580 nm. Orlistat was used as a positive control of inhibition.

#### 4.8.3. Angiotensin Conversing Enzyme I (ACE) activity

The activity of ACE was measured according to Hou et al. [66], with slight modifications. In a 96-well quartz plate, 30 µL of extract (dissolved in Tris buffer 50 mM, pH 7.5, containing 300 mM of NaCl) were mixed with 150 µL of 1.0 mM of FAPGG in the same Tris buffer. The reaction was initiated by adding 10 µL of ACE solution in deionized water. The plate was incubated for 30 min at 37 °C and the absorbance was measured every 60 s at 340 nm. Captopril was used as a positive control of inhibition.

### 4.9. Statistical Analysis

All experiments were performed with at least three independent assays. Data were statistically analyzed by a trial version of GraphPad Prism 6.01 software (OriginLab Corporation, Northampton, MA, USA) using one-way ANOVA analysis and Tukey’s multiple comparisons test.

## 5. Conclusions

This study showed the potential of four seaweeds prevalent in Europe, and their extracts, as natural options for improving, simultaneously, the nutritional and health value of foods. To the best of our knowledge, this is the first study demonstrating that the same seaweed/extract can be used in the food industry to replace synthetic additives (particularly extracts of *F. vesiculosus* as an antioxidant), improve nutritional value (more specifically, due to the high fiber content of all studied seaweeds and interesting concentrations of several important minerals) and contribute to reducing the impact of pathologies associated with the modern life style (especially *F. vesiculosus* extracts for α-glucosidase inhibition and *U. rigida*, *Gracilaria* sp. and *S. latissima* extracts for ACE inhibition).

## Figures and Tables

**Table 1:** Nutritional value of the four seaweeds.

Total Content (g/100 g Seaweed dw)	Sample			
	*U. rigida*	*Gracilaria* sp.	*F. vesiculosus*	*S. latissima*
**Total Carbohydrates**	58.1 ± 0.7 ^b^	46.9 ± 0.4 ^d^	56.4 ± 0.4 ^c^	68.9 ± 0.3 ^a^
**Fiber**				
**Total**	36.6 ± 1.5 ^b^	40.6 ± 3.8 ^a,b^	45.0 ± 0.1 ^a^	40.9 ± 0.6 ^a^
**Soluble**	17.7 ± 1.1 ^b^	22.8 ± 1.3 ^a^	7.6 ± 0.3 ^d^	12.8 ± 0.3 ^c^
**Insoluble**	18.9 ± 0.8 ^c^	17.9 ± 3.3 ^c^	37.4 ± 0.3 ^a^	28.2 ± 0.3 ^b^
**Ash**	31.7 ± 0.6 ^a^	28.9 ± 0.2 ^b^	25.5 ± 0.2 ^c^	20.4 ± 0.1 ^d^
**Minerals (mg/100 g dw)**				
**Na**	2424.0 ± 233.8 ^b^	1594.7 ± 296.6 ^c^	2266.1 ± 35.5 ^b^	3048.3 ± 129.4 ^a^
**K**	2466.6 ± 65.2 ^c^	9243.1 ± 205.8 ^a^	4083.1 ± 61.0 ^b^	3869.4 ± 138.7 ^b^
**Ca**	414.3 ± 33.8 ^c^	200.4 ± 24.3 ^d^	1382.0 ± 5.1 ^a^	919.4 ± 32.5 ^b^
**Mg**	3758.6 ± 430.3 ^a^	285.7 ± 59.5 ^b^	835.5 ± 9.7 ^a^	611.1 ± 25.0 ^b^
**Fe**	110.2 ± 22.6 ^b^	211.0 ± 52.3 ^a^	8.8 ± 1.1 ^c^	185.4 ± 8.2 ^a^
**Mn**	6.84 ± 1.05 ^c^	15.66 ± 2.85 ^b^	54.66 ± 0.31 ^a^	0.56 ± 0.02 ^d^
**Cu**	3.27 ± 0.26 ^b^	3.46 ± 0.49 ^b^	3.05 ± 0.04 ^b^	3.86 ± 0.18 ^a^
**Zn**	3.27 ± 0.26 ^a^	3.45 ± 0.61 ^a^	3.06 ± 0.06 ^a^	3.86 ± 0.18 ^a^
**Ni**	1.07 ± 0.09 ^c^	1.93 ± 0.43 ^b^	2.49 ± 0.08 ^a^	0.26 ± 0.02 ^d^
**Protein**	9.3 ± 0.5 ^c^	23.6 ± 0.2 ^a^	15.1 ± 0.2 ^b^	10.2 ± 0.3 ^c^
**Lipid**	0.9 ± 0.1 ^b^	0.7 ± 0.1 ^b,c^	3.0 ± 0.3 ^a^	0.5 ± 0.1 ^c^
**Caloric content (kcal/100 g dw)**	167.3	112.7	162.6	198.3

Different letters in the same row indicate significant differences (*p* < 0.05).

**Table 2:** Major lipid components of the four seaweeds.

Compound	Compound Content (mg/kg dw)			
	*U. rigida*	*Gracilaria* sp.	*F. vesiculosus*	*S. latissima*
***Saturated Fatty Acids***				
Myristic acid	77.9 ± 34.7 ^b^	100.5 ± 49.5 ^b^	927.9 ± 114.6 ^a^	185.2 ± 12.5 ^b^
Palmitic acid	595.3 ± 67.8 ^b^	458.1 ± 146.0 ^b,c^	1298.1 ± 106.2 ^a^	361.3 ± 22.2 ^c^
Stearic acid	85.6 ± 17.0 ^c^	98.4 ± 1.9 ^b,c^	539.9 ± 37.9 ^a^	146.4 ± 4.3 ^b^
***Unsaturated Fatty Acids***				
Palmitoleic acid	86.4 ± 37.2 ^b^	39.8 ± 14.2 ^b^	154.8 ± 19.8 ^a^	60.4 ± 6.0 ^b^
Oleic acid	410.2 ± 60.7 ^b^	136.7 ± 7.7 ^c^	756.1 ± 117.7 ^a^	134.4 ± 16.4 ^c^
Linoleic acid	97.4 ± 24.1 ^c^	59.9 ± 14.5 ^c^	815.0 ± 45.2 ^a^	240.3 ± 11.7 ^b^
α-Linolenic acid	261.9 ± 31.3 ^b^	216.2 ± 48.3 ^b^	1760.7 ± 151.0 ^a^	385.0 ± 13.1 ^b^
Eicosapentaenoic acid	v	61.9 ± 7.3 ^c^	276.8 ± 15.7 ^a^	111.5 ± 5.3 ^b^
***Esters of Fatty Acids***				
Butyl octadecatetraenoate	160.8 ± 42.2 ^a^	40.3 ± 6.8 ^b^	182.4 ± 14.1 ^a^	61.5 ± 4.7 ^b^
***Long Chain Alcohols***				
2-Hexadecen-1-ol	85.5 ± 4.4 ^b^	22.9 ± 18.2 ^c^	123.8 ± 20.3 ^a^	14 ± 2.3 ^c^
***Diterpenes***				
Phytol	30.8 ± 11.1 ^c^	279.7 ± 24.8 ^a^	115.6 ± 16.8 ^b^	16.6 ± 3.0 ^c^
Neophytadiene	172.3 ± 7.6 ^a^	39.5 ± 9.7 ^b^	182.5 ± 35.3 ^a^	27.7 ± 2.8 ^b^
***Sterols***				
Cholesterol derivatives	87.8 ± 10.0 ^b^	888.1 ± 185.6 ^a^	921.2 ± 136.9 ^a^	202.3 ± 24.3 ^b^
∑ Saturated fatty acids	758.8 ± 119.5 ^b^	657.0 ± 197.4 ^b^	2765.9 ± 257.7 ^a^	692.9 ± 39.0 ^b^
∑ Unsaturated fatty acids	855.9 ± 153.3 ^b,c^	514.5 ± 92.0 ^c^	3763.4 ± 349.4 ^a^	931.6 ± 52.5 ^b^
∑ Ω3	261.9 ± 31.3 ^c^	278.1 ± 55.6 ^b,c^	2037.5 ± 166.7 ^a^	496.5 ± 18.4 ^b^
∑ Ω6	97.4 ± 24.1 ^c^	59.9 ± 14.5 ^c^	815.0 ± 45.2 ^a^	240.3 ± 11.7 ^b^
Ω6/Ω3	0.37	0.22	0.40	0.48
∑ Sterols	87.8 ± 10.0 ^b^	888.1 ± 185.6 ^a^	921.2 ± 136.9 ^a^	202.3 ± 24.3 ^b^

V, vestigial; Different letters in the same row indicate significant differences (*p* < 0.05).

**Table 3:** Total phenolic content (TPC), antioxidant activity measured by 2,2-diphenyl-1-picrylhydrazyl (DPPH) and ferric reducing antioxidant power (FRAP) methods.

Sample	Extract	Yield (g/100 g Seaweed dw)	TPC (g GAE/100 g Extract)	DPPH (g AAE/100 g Extract)	FRAP (g BHAE/100 g Extract)
***U. rigida***	**RTWE**	43.1 ± 0.7 ^b^	0.23 ± 0.03 ^b^	nd	0.08 ± 0.02 ^d^
	**HWE**	46.2 ± 0.6 ^a^	0.16 ± 0.02 ^b^	nd	0.17 ± 0.03 ^c^
	**Eth80**	18.9 ± 1.8 ^c^	0.53 ± 0.07 ^a^	0.09 ± 0.01 ^a^	0.67 ± 0.04 ^a^
	**Acet70**	16.2 ± 0.4 ^c^	0.48 ± 0.06 ^a^	0.08 ± 0.01 ^a^	0.53 ± 0.03 ^b^
***Gracilaria* sp.**	**RTWE**	30.7 ± 1.4 ^a^	0.59 ± 0.03 ^a^	nd	nd
	**Eth80**	23.3 ± 0.8 ^b^	0.49 ± 0.03 ^b^	0.04 ± 0.01 ^a^	0.23 ± 0.02 ^a^
	**Acet70**	23.2 ± 1.6 ^b^	0.54 ± 0.01 ^a,b^	0.04 ± 0.00 ^a^	0.21 ± 0.03 ^a^
***F. vesiculosus***	**RTWE**	27.8 ± 2.3 ^a^	1.48 ± 0.11 ^c^	0.57 ± 0.06 ^c^	2.90 ± 1.02 ^b^
	**HWE**	30.5 ± 0.8 ^a^	1.74 ± 0.09 ^c^	0.77 ± 0.12 ^c^	3.23 ± 1.00 ^b^
	**Eth80**	15.3 ± 1.8 ^b^	5.66 ± 0.26 ^a^	2.60 ± 0.28 ^a^	8.30 ± 1.04 ^a^
	**Acet70**	18.2 ± 0.9 ^b^	3.91 ± 0.09 ^b^	1.47 ± 0.12 ^b^	8.03 ± 3.91 ^a^
***S. latissima***	**RTWE**	40.2 ± 1.6 ^a,b,c^	0.47 ± 0.02 ^b^	0.20 ± 0.04 ^a^	0.68 ± 0.02 ^c^
	**HWE**	44.3 ± 0.9 ^a^	0.78 ± 0.06 ^a^	0.25 ± 0.05 ^a^	1.06 ± 0.06 ^a^
	**Eth80**	36.5 ± 2.2 ^b,c^	0.19 ± 0.02 ^c^	0.07 ± 0.02 ^b^	0.21 ± 0.02 ^d^
	**Acet70**	37.5 ± 2.8 ^b^	0.52 ± 0.02 ^b^	0.25 ± 0.01 ^a^	0.87 ± 0.07 ^b^

nd—not detected. Different letters in the same column (for each seaweed) indicate significant differences (*p* < 0.05). Hot water extraction (HWE) of *Gracilaria* sp. was not obtained due to its high agar content which limits processing.

**Table 4:** Inhibitory activity of seaweed extracts, and of reference compounds, towards α-glucosidase, lipase and angiotensin converting enzyme (ACE).

Sample		α-Glucosidase		Lipase		ACE	
		IC_50_	%inb	IC_50_	%inb	IC_50_	%inb
***Seaweed extract***							
*U. rigida*	RTWE	-	ni	-	ni	-	24.7 ± 5.0% (4.6)
	HWE	-	ni	-	ni	-	24.3 ± 7.1% (4.8)
	Eth80	-	ni	-	ni	3.67 ± 0.14 ^b^	-
	Acet70	-	ni	-	ni	4.33 ± 0.30 ^a^	-
*Gracilaria* sp.	RTWE	-	ni	-	ni	-	15.8 ± 4.3% (4.7)
	Eth80	-	ni	-	26.1± 3.7% (3.0)	3.21 ± 0.23 ^a^	-
	Acet70	-	ni	-	ni	3.45 ± 0.26 ^a^	-
*F. vesiculosus*	RTWE	6.73 ± 0.74 ^c^	-	-	25.0 ± 2.8% (3.0)	-	ni
	HWE	5.12 ± 0.81 ^b^	-	-	15.2 ± 2.5% (3.0)	-	ni
	Eth80	0.119 ± 0.036 ^b^	-	-	ni	-	ni
	Acet70	0.032 ± 0.003 ^a^	-	-	19.5 ± 3.1% (3.0)	-	ni
*S. latissima*	RTWE	6.73 ± 0.74 ^c^	-	-	ni	4.48 ± 0.66 ^b^	-
	HWE	5.12 ± 0.81 ^b^	-	-	ni	-	39.6 ± 2.3% (4.7)
	Eth80	-	47.8 ± 4.5% (7.3)	-	30.8 ± 4.1% (3.0)	4.56 ± 0.05 ^a^	-
	Acet70	1.68 ± 0.22 ^a^	-	-	ni	3.98 ± 0.29 ^b^	-
***Reference compound***							
Acarbose		0.264 ± 0.041	-	-	-	-	-
Orlistat		-	-	0.075 ± 0.004	-	-	-
Captopril		-	-	-	-	7.8 × 10^−7^ ± 1.1 × 10^−7^	-

Values of inhibition of seaweed extracts are expressed as IC_50_ (in cases where at least 50% of inhibition was achieved for the maximum tested concentrations) or as % of inhibition (in cases where 50% of inhibition was not achieved for the maximum tested concentration). IC_50_ values of seaweed extracts and reference compounds are expressed as mg/mL. %inb—% inhibition of extract, expressed in %, observed for the maximum tested concentration (value in parenthesis); “-“—not determined; ni—no inhibition. HWE of *Gracilaria* sp. was not obtained due to its high agar content which limits processing. Different letters in the same column (for each seaweed) indicate significant differences (*p* < 0.05).

**Table 5:** Nutritional values of seaweed extracts.

Seaweed	Extract	g Ash/100 g ext_dw_	g GlcE/100 g ext_dw_	g Sol Fiber/100 g ext_dw_	g Protein/100 g ext_dw_
***U. rigida***	**RTWE**	48.5 ± 0.7 ^b^	19.6 ± 0.6 ^b^	33.8 ± 3.0 ^a^	5.2 ± 0.1 ^c^
	**HWE**	49.2 ± 0.5 ^b^	19.4 ± 1.2 ^b^	37.3 ± 1.1 ^a^	3.9 ± 0.3 ^d^
***Gracilaria* sp.**	**RTWE**	55.9 ± 1.0 ^a^	8.9 ± 1.5 ^e^	14.9 ± 0.6 ^d^	19.0 ± 0.1 ^a^
***F. vesiculosus***	**RTWE**	28.8 ± 0.3 ^c^	7.5 ± 1.0 ^e^	17.2 ± 0.5 ^c^	7.0 ± 0.5 ^b^
	**HWE**	29.4 ± 0.4 ^c^	13.8 ± 2.1 ^d^	24.5 ± 2.8 ^b^	6.6 ± 0.4 ^b^
***S. latíssima***	**RTWE**	29.7 ± 0.4 ^c^	18.2 ± 0.7 ^b^	17.1 ± 0.9 ^c^	4.4 ± 0.1 ^d^
	**HWE**	27.8 ± 0.1 ^c^	23.4 ± 3.1 ^a^	25.2 ± 0.2 ^b^	4.6 ± 0.2 ^d^

Different letters in the same column (for each seaweed) indicate significant differences (*p* < 0.05). GlcE-glucose equivalents.
